# Barriers to accessing health care among young people in 30 low‐middle income countries

**DOI:** 10.1002/hsr2.733

**Published:** 2022-07-20

**Authors:** Nitish Nachiappan, Shona Mackinnon, Jean P. Ndayizeye, Geva Greenfield, Dougal Hargreaves

**Affiliations:** ^1^ School of Public Health Imperial College School of Medicine London UK; ^2^ Division of Basic Medical Sciences University of Global Health Equity Kigali Rwanda

**Keywords:** adolescent health, global health, healthcare barriers, healthcare access

## Abstract

**Background:**

Previous studies focusing on high‐income countries have shown that young people often face greater barriers to accessing healthcare than older adults. However, in low‐middle income countries (LMICs), there have been a paucity of cross‐country, quantitative studies highlighting these barriers.

**Aim:**

This exploratory study aims to provide a scoping review of the publicly available Demographic and Heath Survey (DHS) data with a view to form the basis for further work.

**Materials and methods:**

Data on insurance coverage, agency, and access to evidence‐based family planning from 30 countries in the DHS were compared between age groups. Data on 586,250 participants 15–24 years (33% male) and 854,660 participants 25–49 years (16% male) from 30 LMICs were analyzed.

**Results:**

Significantly greater barriers to accessing healthcare were observed across six variables in younger population when compared to older adults across all survey questions with an average of 8.4% point difference. Also, there was wide country‐level variation: the maximum differences between age groups were 33% points; Rwanda was the only country with no age differences.

**Discussion:**

This study highlights several possible themes for future research into improving access to healthcare for young people. These themes include more detailed evaluation of country‐specific policies to reduced barriers to healthcare for young people and further research into the causative factors that can influence healthcare utilization by young people.

**Conclusion:**

Our analysis showcases increased barriers to healthcare access for young people in LMICs. We argue that they can only be improved by targeted policies and direct community engagement.

## 1 BACKGROUND

Providing accessible, high‐quality health services for young people is an important investment in a country's future health and economic prosperity.^1^ Globally there are 1.8 billion young people, of which 90% live in low‐middle income countries (LMICs). There are many ways to categorize this group such as youths (age 15–24) and adolescents (age 10–19).^2^ However, within this report these groups will be referred using the encompassing term “young people” (age 10–24) unless stated otherwise.

Even though there is clear evidence of benefits of focusing health interventions in this group, they have often been neglected by our health systems. An example of this inequality can be seen in the rates of mortality decline within different age groups. A robust cross‐country study identified that mortality in infants (historically identified as a vulnerable group, aged [1–5]) has fallen by 75% since 1980. However, during the same time period, the mortality of young people (10–24) fell significantly less in the same countries.^3^ This resulted in young people having a higher mortality than infants in some countries, which reflects the relative lack of policy attention on the health of young people.

A major reason for these poor health outcomes can be attributed to the fact that young people are less likely to access healthcare compared to other age groups, and present later when they seek care which results in poor prognosis.^4^ Furthermore, qualitative literature across many countries show that young people typically report poorer experiences when they access healthcare compared to other groups.^5^, ^6^

A healthcare barrier is something that restricts the use of a health service, either through access or utilization.^7^ These can be studied through two main methods; poor utilization of the service or reports of unmet need through surveys. Measuring poor utilization of a service tends to capture more of healthcare barrier trends, however this is a proxy measure and therefore can introduce unseen confounding variables. Unmet need reporting specifically relates to healthcare barriers and therefore provides a good indicator but can be specific and therefore miss unreported barriers and general trends. Therefore, it is appropriate to use a two‐pronged approach encompassing both methods when measuring healthcare barriers to reduce the individual limitations.

Current literature indicates that this may be due to health services not meeting the specific needs of this age group, and this group facing additional barriers to accessing healthcare.^5^, ^6^, ^8^ This is supported by the fact that unmet healthcare needs in young people are known to be associated with poor health outcomes.^8^, ^9^ An American study showed that individuals who had unmet needs in their adolescence were up to 52% more likely to have poorer health outcomes compared to matched individuals who did not.^5^ Through this study, it is evident that healthcare barriers have a key role in reducing contemporary health outcomes.

Published reports clearly indicate that the unmet need for healthcare and receipt of low‐quality healthcare in adolescence are important predictors of poor health outcomes in adulthood.^3^, ^5^, ^10^, ^11^ Furthermore, poor health outcomes of this group are well documented in qualitative literature,^12^ and evidence suggests that a contributing factor to this is due to this group facing additional healthcare barriers compared to other age groups.^4^, ^11^ Previous literature also indicates that healthcare barriers for young people exist around the cost of healthcare, lack of health insurance, and minimal empowerment to make independent decisions about their care. Existing literature also suggests that care quality is often variable when they can access it.^9^, ^10^, ^11^ Tackling these barriers could make an important contribution in improving young people's health as well as reducing the poorer quality care that this group receives compared to other age groups.

There is a significant body of literature across many LMICs which describe the presence of barriers to healthcare. These relate both to young people^12^, ^13^ and older adults.^14^, ^15^ However, the Lancet commission on adolescents identified the lack of quantitative data looking at the prevalence of healthcare access for young people in LMICs as a substantial research gap.^1^ Cross‐country quantitative data that builds upon the body of qualitative research is essential to make meaningful improvements to the health of young people in LMICs. Sociocultural and economic differences must be considered when using these papers to guide research in the LMIC population, hence there is a clear need for quantitative research drawn from young people living in LMICs.

The purpose of this exploratory work is to highlight the existence of these barriers using publicly available Demographic and Health Surveys (DHS) dataset to form the basis for further work. Using variables that are comparable to those used within the published literature, this study intends to provide a basis for policy intervention in these countries. We performed an exploratory cross‐country quantitative analysis of the prevalence of healthcare barriers amongst young people (15–24) and older adults (25–49) across 30 LMICs to identify if young people face additional healthcare barriers compared to older adults.

## 2 DESCRIPTION OF THE DATASET

We accessed data from the DHS website (https://www.statcompiler.com/en/) and compared the prevalence of healthcare barriers in different age groups by country, using the most recent available survey (Supporting Information: Appendixes 1 and 2). Ethical review for this study was not needed as the DHS is an anonymised, open‐access database.

The DHS program has a rigorous survey method which selects all appropriate individuals through a two‐stage survey process and also has high response rates (95%+) across all countries sampled. DHS methodology uses a mixture of survey tools including four model questionnaires including separate surveys for men, women, and household, as well as biomarker collection. Methods are uniform and standardized across countries and survey years, allowing direct comparisons to be made between countries. All surveys were nationally representative and had large enough sample sizes to allow meaningful comparisons between age groups. Definitions of the study variables and wider description of the data is provided in Supporting Information: Material 2. We would like to highlight that there have been some changes in the definitions of unmet need as the survey methodology evolved. However, these have been accounted for within the dataset.

## 3 INFORMATION SYNTHESIS

Previous literature in this field was reviewed, with a focus on cross country studies that compared healthcare access between younger and older adults or between adolescents in different countries. This was conducted to find methods to identify unmet need and poorer health outcomes and build a conceptual framework to search the DHS database as a part of this exploratory phase. Based on this conceptual framework, we identified the survey questions which were adapted from the World Health Organization accelerated action for the health of adolescents action plan, informed by previous work.^5^, ^16^ We summarized the main barriers to healthcare access in a population into four main categories: access issues related to cost, barriers due to nonfinancial factors, low perceived importance of the problem, and perceived negative consequences of accessing healthcare.

The DHS database was systematically searched to find survey questions and countries. Data extraction from the DHS database was conducted using the DHS STATcompiler program. The extracted data were then cleaned and standardized for analysis. The average household response rate to survey questions across all countries was 97%,^17^ and the average women response rate was 96%.

The inclusion criteria for a survey question was to meet one aspect of the conceptual framework and to have age disaggregation between young people (10–24) and an older age group (25+). Any country selected needed to have data on at least half of the questions during the period of 2005–2018. Applying this selection criteria left six questions and 30 countries (full list of these questions and countries are provided in the Supporting Information).

A healthcare barrier was defined as something that restricts the use of a health service and/or reflects lack of access to evidence‐based care. We selected six variables which reflected such barriers and had high levels of complete data across a wide range of countries:

- Final say in own healthcare (women)*.
- Final say on own healthcare (men)*.
- Unmet need for family planning*.
- Demand for family planning satisfied by modern methods*.
- No health insurance (men).
- No health insurance (women).

*Denotes that questions were only addressed to married participants.

The data present in the DHS database was gender specific and was provided in the groups of “all men,” “all women,” “married men,” and “married women.” The DHS data were disaggregated into 15–24, 25–34, and 34–49 age bands. The encompassing term “young people” was used to describe the 15–24 category for the analysis of data, to keep consistency with literature. The “all men” category also included 50+ as part of the age disaggregation; however, the DHS database provided a 15–49 total, hence the 50+ category was excluded to increase comparability between different questions. All data collected was weighted by the DHS to be nationally representative, to account for the under sampling and oversampling that occurred in geographical regions during the DHS survey. The categories (25–34) and (35–49) were weighted by proportion and aggregated to produce a (25–49) “older adults” category. No other specific data reduction or homogenization techniques were used. For each country, the proportion of respondents in each age group (15–24, 25–49) reporting healthcare barriers were calculated; age groups were then compared using the two‐proportion *z*‐test. Next, data from all countries were aggregated, weighted so that each country contributed equally to the overall proportions. The mean values across all countries for each survey question were calculated and compared between age groups. Comparisons between the two groups (15–24) and (25–49) were conducted using the two‐proportion *z*‐test.^18^ This was selected as the most appropriate statistical test to compare two groups within the DHS as this test is compatible with large population difference between age groups. Furthermore, the essential criteria of this test were that each country was sampled independently and the population of each country was 20 times the sample size, which were met.^19^ Significance was defined as *p* < 0.05. The first set of analyses were standardized so that each country was given equal weight. The (15–24) and (25–49) group were then compared, to look at any differences between younger and older age groups across all questions. Countries were then compared to each other in each respective survey question, to look for differences between countries. To facilitate comparisons between questions that looked at positive and negative outcomes, the questions that showed a negative outcome (no health insurance men, no health insurance women, and unmet need for family planning) were inverted to present the proportion of participants giving a positive response (please refer to Supporting Information: Material 2).

## 4 RESULTS

Data were analyzed on 586,250 participants 15–24 years (33% male) and 854,660 participants 25–49 years (16% male).

**FIGURE 1:**
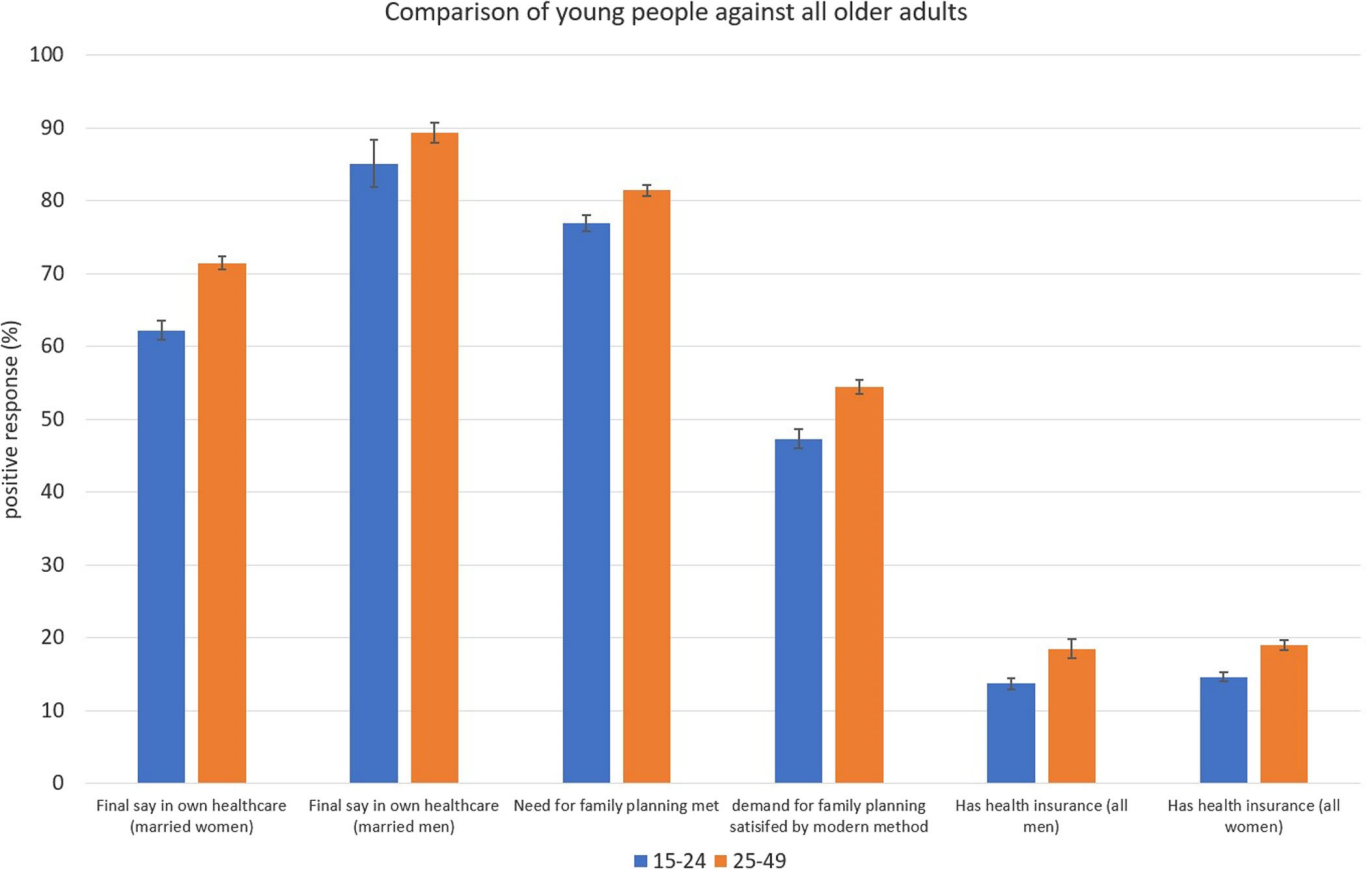
Proportion reporting healthcare barriers among young people (15–24) versus older adults (25–49) in Demographic and Health Surveys from 30 low‐middle income countries. Error bars show 95% confidence intervals.

Figure 1 shows the proportion of participants across all countries that responded positively to each indicator of healthcare access. Young people were significantly less likely to report being able to access healthcare compared to older adults across all questions: “Final say in own healthcare – married women” (62.2% vs. 71.4%, *p* < 0.01), “final say in own healthcare – married men” (85.1% vs. 89.3%, *p* < 0.01), “need for family planning met” (76.9% vs. 81.4%, *p* < 0.01), “demand for family planning satisfied by modern methods” (47.3% vs. 54.4%, *p* < 0.01), “health insurance – all men” (13.7% vs. 18.5%, *p* < 0.01), and “health insurance – all women” (14.6% vs. 19.0%, *p* < 0.01).

When each survey question was analyzed individually, wide variations were seen between countries. For example, the proportion of young women in India who reported being able to access modern methods of family planning was 33% points lower than among older women (43.3% vs. 76.6%, *p* < 0.01). In contrast, Rwanda was one of three countries (along with Congo, Cameroon) where young women had significantly better access than older adults (72.0% vs. 64.9%, *p* < 0.01).

Rwanda was the only country where no significant age differences were found for any survey questions data was present for. Country‐specific results for each question are presented in Supporting Information: Figures [Link], [Link], [Link], [Link], [Link], [Link].

## 5 DISCUSSION

In this pilot study, across 30 LMICs, we found that young people reported more barriers to accessing effective healthcare than older adults. The data support perceptions by young people and healthcare professionals that young people often face additional barriers to accessing healthcare in many LMICs, and echoes findings across high‐income countries (HICs). These trends are seen a variety of countries with different levels of economic development, which indicate that some barriers that young people face may be independent of the economic development of the LMIC. This theory is supported by how Rwanda has $57 health spending per capita but appears to perform better in giving young people equal access healthcare compared to Sao Tome and Principe which spends triple this amount.^20^

**FIGURE 2:**
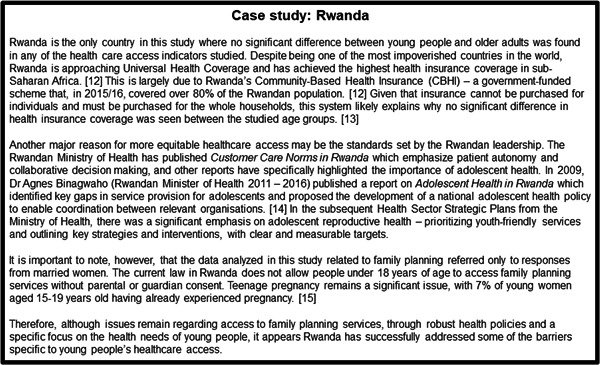
Case study on Rwanda.

These included cost barriers (reflected in health insurance coverage), lack of agency (reflected in the proportion who reported they had the final say in accessing healthcare) and unmet need for modern family planning methods. These findings extend previous qualitative and single‐country studies in LMICs and cross‐country studies in HICs which have also reported more barriers to accessing healthcare among young people. However, the wide variation between countries reinforces the message that such age differences are far from inevitable: they can be mitigated or even avoided by using targeted strategies. Certain countries such as Rwanda had no differences between age groups across multiple survey questions, indicating that healthcare barriers for young people are not inevitable (Figure 2).^21^, ^22^, ^23^, ^24^

Also, young people display country specific health behaviors and attitudes based on a number of ethnographic factors. However, this does not appear to influence the inequalities young people face in healthcare access compared to older adults, with many countries with varying geographic distribution showing similar health barriers. Furthermore, it is interesting to note that the patterns seen are very similar to those seen in young people in HICs, suggesting how this is a worldwide problem.^9^ It also appears that this inequality in healthcare access appears in both genders, however both young and older females faced more barriers to healthcare compared to their male group counterparts.

The DHS survey questions with data availability were focused on married women, with half of the final survey questions focusing only on this subgroup. It is also important to note there is a multiyear gap between surveys in different countries due to data availability, and healthcare barriers could have changed over this period. This means that we cannot infer the magnitude of these barriers young people face in other LMICs. It is also possible that differences between age group and/or countries may reflect different understanding of the question or different expectations of agency, insurance coverage, or access to family planning. Also, the definition for unmet need for family planning was changed over the period of sampling years reported within this paper. However, as outlined within the DHS program website, this has been accounted for and the reported data estimates the total demand for family planning, the satisfied share of this demand and the unmet need for sexually active unmarried women.^25^

In addition to the data available from the DHS, studies such as the Research on Early Life and Aging Trends and Effects^15^, ^26^, ^27^ have compiled cross national data that contains rich and relevant dataset that can help to examine the effects of several health conditions. However, many of the data synthesis and resulting publications focus on older adults and long‐term health conditions such as diabetes. There is a clear need for focusing on early life conditions, which forms the basis for this current report. By focusing on access issues related to cost, barriers due to nonfinancial factors, low perceived importance of the problem and perceived negative consequences of accessing healthcare, the current report, and future work based on these variables can give a surrogate measure of the socioeconomic impact.

Although one could argue that the country selection and the variables used within this study does not provide an overall picture, this report is driven by immediate data availability to highlight the gap in this area. One needs to understand that there are several other available variables in the DHS surveys concerning access to healthcare such as the access to antenatal care, access to institutional delivery and skilled worker, and distance to the healthcare facilities. While these could provide a richer picture there have been gaps across countries and selection dates in these variables.

One of the perceived limitations of the current paper relates to the cluster effect and correlation that may exist among the samples from each specific country. Access to health services across age groups and gender might be different in various countries and one could use mathematical techniques such as generalized linear models models to account for such clustering effects when using merged datasets to compare data between different countries.

The main purpose to explore the validity of the methodological approaches to form the basis of further work. The large differences in responses between countries suggest that there are multiple external factors that affect healthcare barriers, and these can be targeted by health systems. Interventions such as prioritizing youth friendly services as done in Rwanda (Figure 2) provide examples on how we can improve healthcare access for young people and therefore improve their lifelong health outcomes.

## 6 CONCLUSION

This study highlights several possible themes for future research into improving access to healthcare for young people. These themes include more detailed evaluation of country‐specific policies to reduced barriers to healthcare for young people and further research into the causative factors that can influence healthcare utilization by young people.

## AUTHOR CONTRIBUTIONS

**Nitish Nachiappan**: Conceptualization; data curation; investigation; methodology; writing – original draft; and writing – review and editing. **Shona Mackinnon**: Writing – original draft; and writing – review and editing. **Jean P. Ndayizeye**: Writing – original draft; and writing – review and editing. **Geva Greenfield**: Writing – original draft; and writing – review and editing. **Dougal Hargreaves**: Conceptualization; data curation; investigation; methodology; supervision; and writing – review and editing.

## CONFLICT OF INTEREST

The authors declare no conflict of interest.

## TRANSPARENCY STATEMENT

The lead author (NN) affirms that this manuscript is an honest, accurate, and transparent account of the study being reported; that no important aspects of the study have been omitted; and that any discrepancies from the study as planned have been explained.

## Supporting information

Supporting information.

Supporting information.

Supporting information.

Supporting information.

Supporting information.

Supporting information.

Supporting information.

Supporting information.

## Data Availability

Data taken from a publicly available database, no ethical approval needed.
