# Supplementary figures and images for: Barriers to accessing health care among young people in 30 low‐middle income countries
Source: Health Sci Rep. 2022 Jul 20;5(4):e733. doi: 10.1002/hsr2.733 (PMC9297382; doi:10.1002/hsr2.733)

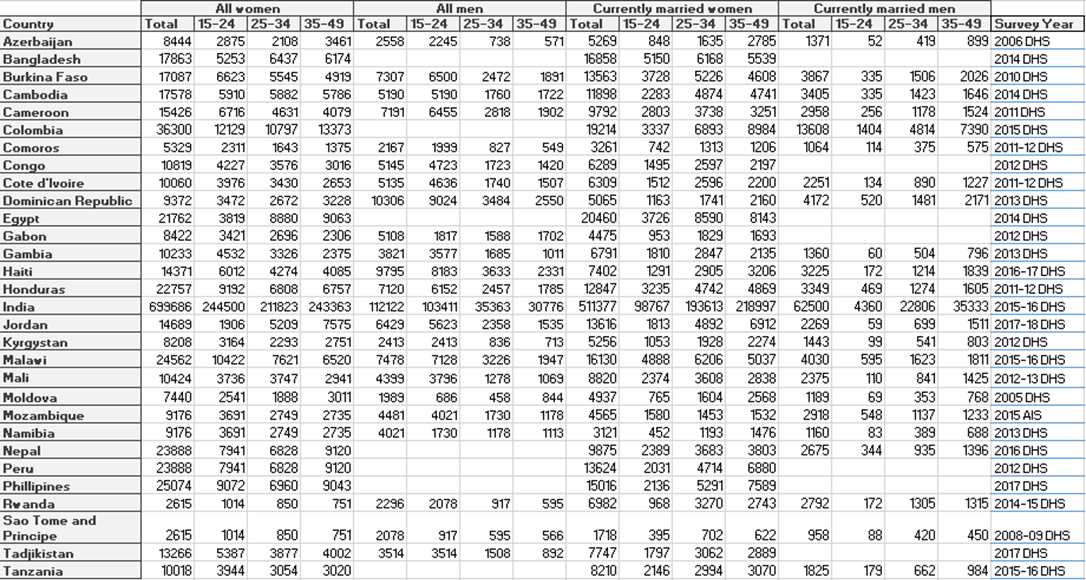

Supplement: Supplementary file 1 — Supporting information. [file HSR2-5-e733-s006.tif]

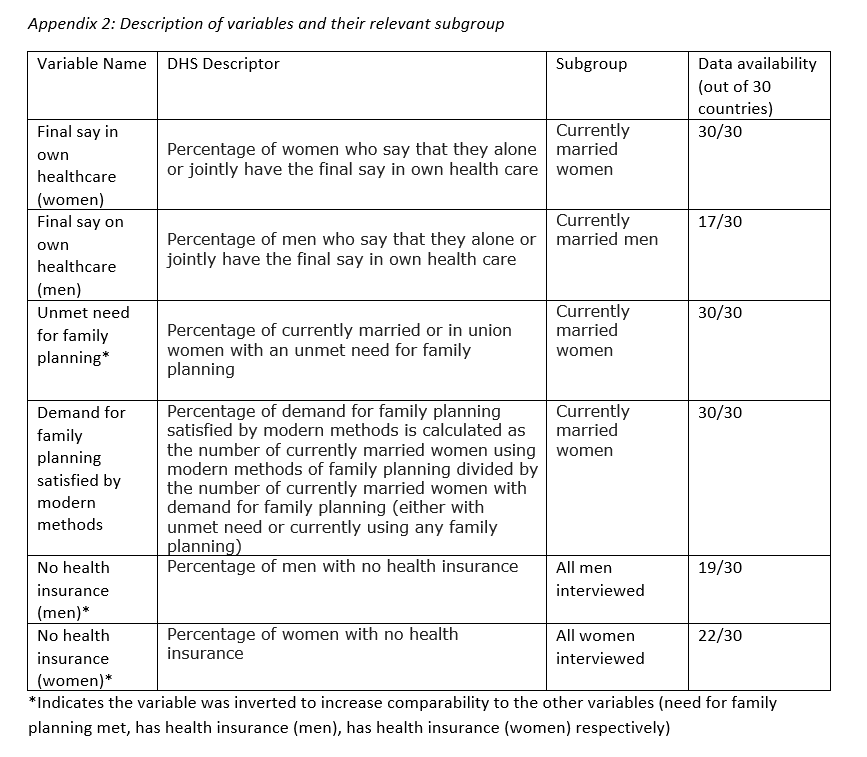

Supplement: Supplementary file 2 — Supporting information. [file HSR2-5-e733-s005.png]

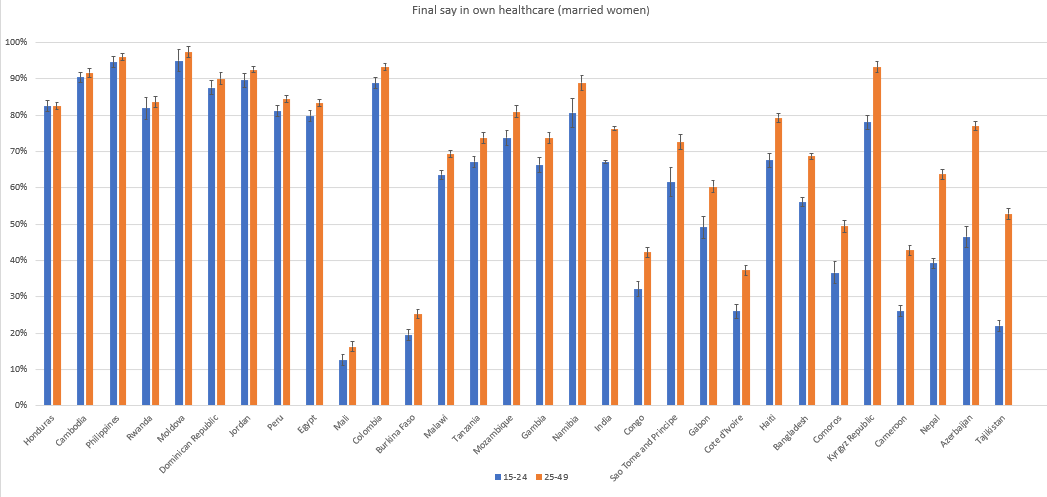

Supplement: Supplementary file 3 — Supporting information. [file HSR2-5-e733-s002.tif]

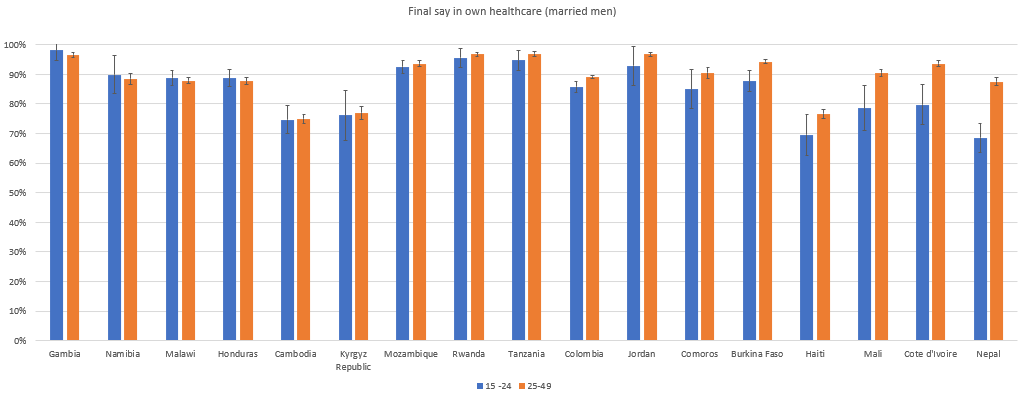

Supplement: Supplementary file 4 — Supporting information. [file HSR2-5-e733-s003.tif]

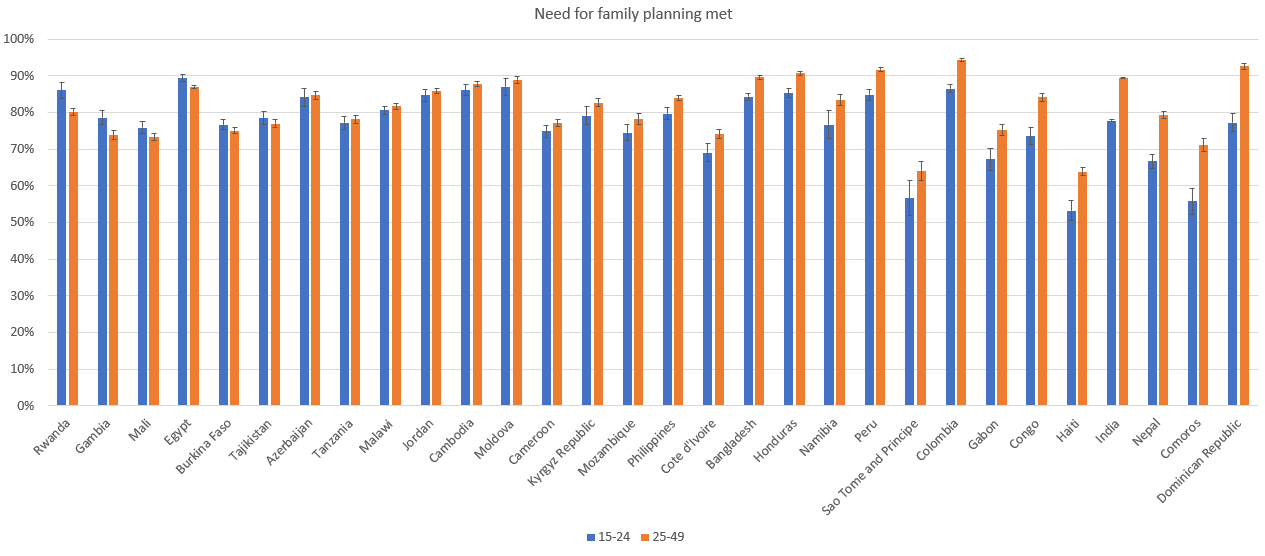

Supplement: Supplementary file 5 — Supporting information. [file HSR2-5-e733-s007.tif]

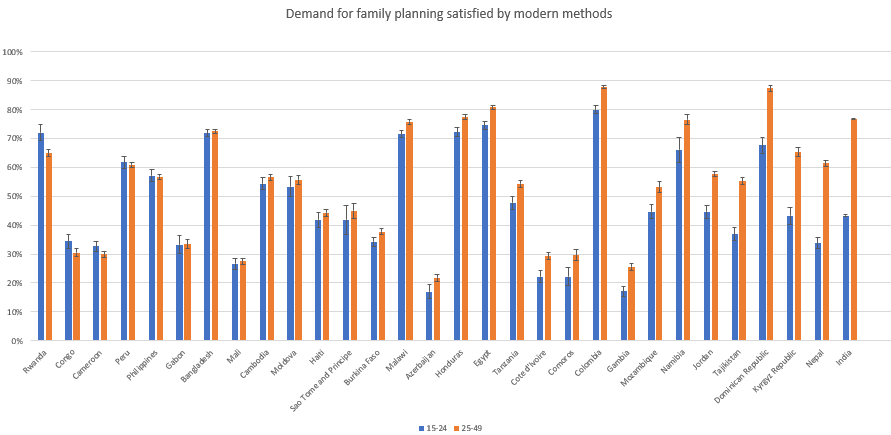

Supplement: Supplementary file 6 — Supporting information. [file HSR2-5-e733-s004.tif]

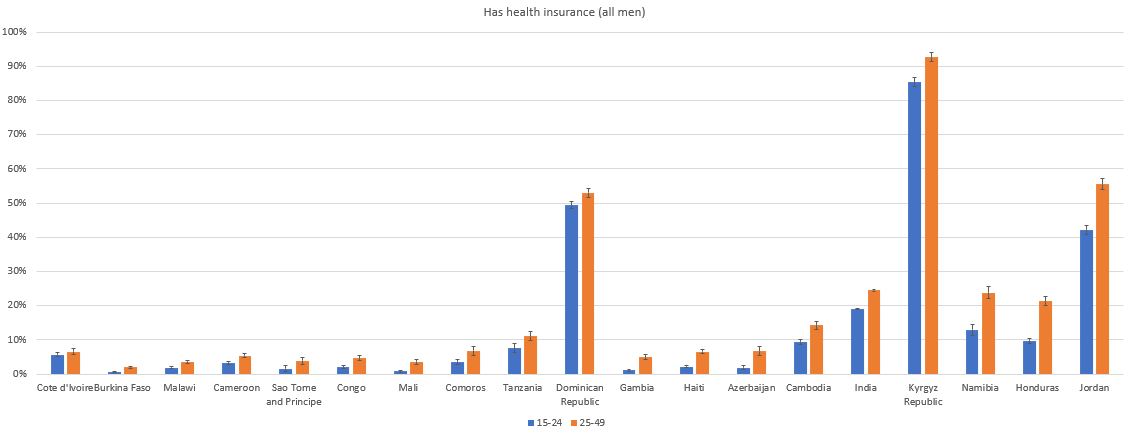

Supplement: Supplementary file 7 — Supporting information. [file HSR2-5-e733-s008.tif]

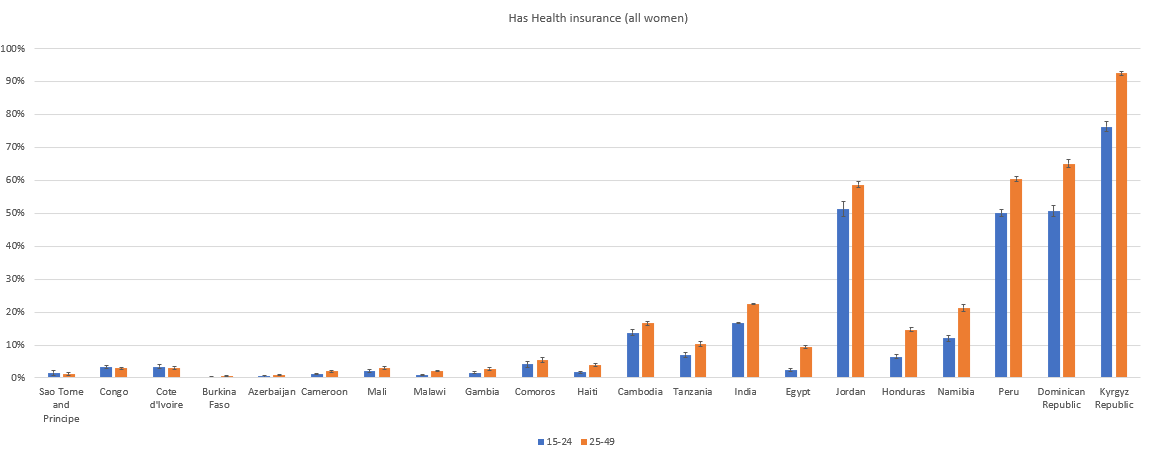

Supplement: Supplementary file 8 — Supporting information. [file HSR2-5-e733-s001.tif]
